# A Chimeric Sudan Virus-Like Particle Vaccine Candidate Produced by a Recombinant Baculovirus System Induces Specific Immune Responses in Mice and Horses

**DOI:** 10.3390/v12010064

**Published:** 2020-01-03

**Authors:** Fangfang Wu, Shengnan Zhang, Ying Zhang, Ruo Mo, Feihu Yan, Hualei Wang, Gary Wong, Hang Chi, Tiecheng Wang, Na Feng, Yuwei Gao, Xianzhu Xia, Yongkun Zhao, Songtao Yang

**Affiliations:** 1 Institute of Military Veterinary Medicine, Academy of Military Medical Sciences, Changchun 130122, China; wufang_0617@163.com (F.W.); Zhang_Shengnan1992@163.com (S.Z.); zhangy99321@163.com (Y.Z.); moruo1996@163.com (R.M.); yanfh1990@gmail.com (F.Y.); whl831125@163.com (H.W.); ch_amms@163.com (H.C.); wgcha@163.com (T.W.); fengna0308@126.com (N.F.); yuwei0901@outlook.com (Y.G.); xiaxzh@cae.cn (X.X.); 2 College of Wildlife Resources, Northeast Forestry University, Harbin 150040, China; 3 Animal Science and Technology College, Jilin Agricultural University, Changchun 130118, China; 4 Key Laboratory of Jilin Province for Zoonosis Prevention and Control, Changchun 130000, China; 5 Jiangsu Co-innovation Center for Prevention and Control of Important Animal Infectious Disease and Zoonoses, Yangzhou 225009, China; 6 College of Veterinary Medicine, Jilin University, Changchun 130062, China; 7 Institute Pasteur of Shanghai, Chinese Academy of Science, Shanghai 20031, China; garyckwong@hotmail.com; 8 Special Pathogens Program, Public Health Agency of Canada, Winnipeg, MB R3E3R2, Canada

**Keywords:** Sudan virus, virus-like particle, vaccine, mice, horse, purified IgG

## Abstract

Ebola virus infections lead to severe hemorrhagic fevers in humans and nonhuman primates; and human fatality rates are as high as 67%–90%. Since the Ebola virus was discovered in 1976, the only available treatments have been medical support or the emergency administration of experimental drugs. The absence of licensed vaccines and drugs against the Ebola virus impedes the prevention of viral infection. In this study, we generated recombinant baculoviruses (rBV) expressing the Sudan virus (SUDV) matrix structural protein (VP40) (rBV-VP40-VP40) or the SUDV glycoprotein (GP) (rBV-GP-GP), and SUDV virus-like particles (VLPs) were produced by co-infection of Sf9 cells with rBV-SUDV-VP40 and rBV-SUDV-GP. The expression of SUDV VP40 and GP in SUDV VLPs was demonstrated by IFA and Western blot analysis. Electron microscopy results demonstrated that SUDV VLPs had a filamentous morphology. The immunogenicity of SUDV VLPs produced in insect cells was evaluated by the immunization of mice. The analysis of antibody responses showed that mice vaccinated with SUDV VLPs and the adjuvant Montanide ISA 201 produced SUDV GP-specific IgG antibodies. Sera from SUDV VLP-immunized mice were able to block infection by SUDV GP pseudotyped HIV, indicating that a neutralizing antibody against the SUDV GP protein was produced. Furthermore, the activation of B cells in the group immunized with VLPs mixed with Montanide ISA 201 was significant one week after the primary immunization. Vaccination with the SUDV VLPs markedly increased the frequency of antigen-specific cells secreting type 1 and type 2 cytokines. To study the therapeutic effects of SUDV antibodies, horses were immunized with SUDV VLPs emulsified in Freund’s complete adjuvant or Freund’s incomplete adjuvant. The results showed that horses could produce SUDV GP-specific antibodies and neutralizing antibodies. These results showed that SUDV VLPs demonstrate excellent immunogenicity and represent a promising approach for vaccine development against SUDV infection. Further, these horse anti-SUDV purified immunoglobulins lay a foundation for SUDV therapeutic drug research.

## 1. Introduction

Ebola virus infections lead to severe hemorrhagic fevers in humans and nonhuman primates, with human fatality rates of up to 90% [1]. The first outbreak of Ebola virus disease occurred in the Republic of Zaire (now the Democratic Republic of the Congo, DRC) and southern Sudan in 1976. From these independent outbreaks, two distinct viruses were identified, Zaire Ebolavirus (EBOV) [2] and Sudan virus (SUDV) [3], which are members of the genus Ebolavirus and have been the cause of sporadic outbreaks in humans throughout the years [1]. Species of Ebolavirus include EBOV, SUDV, Tai Forest Ebolavirus (TAFV), Bundibugyo Ebolavirus (BDBV), Reston Ebolavirus (RESTV) [4]. Both EBOV and SUDV are pathogenic to humans and nonhuman primates, causing severe hemorrhagic fever with high mortality rates of 67–90% [5,6]. Past EBOV outbreaks have been sporadic in nature and confined to central Africa, and thus far, the biggest outbreak on record is the 2013–2016 epidemic in western Africa, with 28,464 cases and 11,323 fatalities [7]. Most of the cases were in Guinea, Liberia, and Sierra Leone, but some of them were imported into Europe and the United States (https://app.who.int/ebola/current-situation/ebola-situation-report-30-march-2016). Currently, the latest devastating outbreak of the Ebola virus disease in the DRC is ongoing. As of today, a total of 3250 cases were reported, including 3133 confirmed and 117 probable cases, of which there have been 2174 fatalities with a mortality rate of 67% (WHO situation report https://www.who.int/csr/don/24-october-2019-ebola-drc/en/). 

The Ebola virus is a non-segmented negative-strand RNA virus belonging to the family Filoviridae and the genus *Ebolavirus* [8]. The genome of filoviruses consists of a single-strand, negative-sense RNA genome of approximately 19 kb length, encoding the following genes in the following orientation: 3′-nucleoprotein (NP)-polymerase cofactor (VP35)-matrix protein (VP40)-glycoprotein (GP)-soluble GP (sGP)-small soluble GP (ssGP)-transcription activator (VP30)-minor matrix protein (VP24)-RNA dependent RNA polymerase (L)-5′ [9]. GP, the critical target antigen that is expressed on the surface of mature virions, is responsible for mediating cell attachment and viral entry [10]. Several vaccine platforms have been reported for EVD vaccine research, including vesicular stomatitis virus (VSV) [11,12], DNA replication-defective adenovirus vectors (Adv) [13,14], human parainfluenza virus type 3 [15], rabies virus [16,17], cytomegalovirus [18], Venezuelan equine encephalitis virus (VEEV) replicons [19] and virus-like particles (VLPs) [20]. There are eight vaccine candidates currently in human clinical trials. The two most promising preclinical vaccine candidates, VSV-EBOV [21] and chAd-EBOV, are in phase 3 clinical trials. No effective treatment for EVD is commercially available; however, China and Russia were the first to license EBOV vaccines in 2018 [22]. At the same time, passive immunotherapy with sera of animal origin has been used for over 120 years to treat bacterial and viral infections and drug intoxications. Currently, there are many therapeutic antibody drugs for treating Ebola virus disease, such as ZMappTM [23] and immunoglobulin F(ab′) 2 fragment [24]. 

VLPs represent a promising vaccine platform for a diverse array of viruses that include influenza virus, rotoviruses, noroviruses, HIV, hepatitis B virus, parvoviruses, rift valley fever virus, human papillomavirus and filoviruses [25,26,27]. VLPs are assembled by one or several proteins, with the distinct advantage of being noninfectious because they lack the viral genome required for replication. VLPs are highly ordered compounds similar to an actual live virus in terms of structure and size. The granular structure of VLPs is beneficial for antigen presentation and cell uptake, which can stimulate powerful innate and adaptive immune responses [28]. VLPs have the advantages of rapid production in large quantities and can generate robust innate, humoral and cellular immunity in animals and humans [29]. Furthermore, pre-existing immunity associated with live carrier vaccines is not hindered by VLP-based immunizations. Previous findings showed that SUDV VLPs could be readily assembled by the co-expression of insect cells with baculoviruses expressing GP, NP, and VP40 [30].

There are currently no approved specialized drugs or vaccines to protect against SUDV disease outbreaks, and thus there is an urgent need for the development of an efficacious, safe and economically viable vaccine or therapeutic antibody to control SUDV infections. Here, we report that production of SUDV VLPs has been accomplished in insect cells by the co-infection with recombinant baculoviruses rBV-GP-GP and rBV-VP40-VP40, and evaluate the ability of SUDV-VLPs to induce SUDV-specific humoral and cellular immune responses in vaccinated mice. Further, horses were immunized with SUDV VLPs, and horse serum was purified to prepare purified immunoglobulins and the purified immunoglobulins had neutralizing activity.

## 2. Materials and Methods 

### 2.1. Cells and Animals

The 293T and Huh7 cells (ATCC) were cultured in Dulbecco’s modified Eagle’s medium (DMEM; Corning-Costar, Coring, NY, USA) supplemented with 10% fetal bovine serum and penicillin-streptomycin (FBS; Gibco, Grand Island, NY, USA). These cells were maintained at 37 °C. The Spodoptera frugiperda (Sf9) cells (Invitrogen, San Diego, CA, USA) were cultured in suspension in SF-900 II serum-free medium (Invitrogen, San Diego, CA, USA) supplemented with 10% FBS and penicillin-streptomycin. These cells were maintained at 27 °C.

Female BALB/c mice, 6–8 weeks old, were purchased from Changchun Institute of Biological Products (Changchun, China). All experiments involving mice adhered to the principles of the Welfare and Ethics Committee of the Military Veterinary Research Institute at the Academy of Military Sciences. The mice were provided ad libitum access to sterilized water and food throughout the study and were vaccinated under BSL-2 conditions. 

Healthy male horses 2–6 years old and 400–500 kg were provided by Red Hill Military horse farm (Changchun, China). Horse studies were conducted with prior approval from the Animal Welfare and Ethics committee of the Institute of Military Veterinary, Academy of Military Medical Sciences (permit number SCXK-2012-017), according to Horse Quarantine and Immunization Protocols for Equine Serum Production.

### 2.2. Construction of Recombinant Baculoviruses

The cloning and construction of the recombinant bacmids, pFastBacDual-GP-GP, and pFastBacDual-VP40-VP40, were carried out. Briefly, we synthesized the full-length GP and VP40 genes (Supplementary material) according to nucleotide frequency (SUDV GP genbank accession no. AY729654.1, AY344234.1, KR063670.1, KU182912.1, NC006432.1, KC545392.1, KC545391.1, KC545390.1, KC545389.1, JN638998.1, KC589025.1, EU338380.1, U23069.1, KC242783.2, KT878488.1, FJ968794.1) (SUDV VP40 genbank accession no. KC545390.1, KC545389.1, KC545391.1, KC545392.1, JN638998.1, NC006432.1, AY729654.1, KU182912.1, KR063670.1, KC589025.1, KT878488.1, KC242783.2, FJ968794.1, KT750754.1, EU338380.1). Two identical full-length GP genes inserted into the pFastBacDual vector (Invitrogen, San Diego, CA, USA) by using KpnI, SmalI, EcoRI and XbaI, the GP genes were under the control of P10 and polyhedron promoters, generating the recombinant plasmid pFastBacDual-GP-GP (Supplementary Figure S1A). The recombinant plasmid pFastBacDual-VP40-VP40 (Supplementary Figure S1B) was constructed using the same strategy. Recombinant plasmids were used to transform *E. coli* DH10Bac competent cells to generate recombinant bacmids. Sf9 cells were transfected with recombinant bacmids using Cellfectin^®^ II Reagent (Thermo Scientific, Carlsbad, CA, USA) according to the manufacturer’s instructions. Transfected cells were incubated at 27 °C for 4 d and harvested, the recombinant baculoviruses rBV-GP-GP and rBV-VP40-VP40 by collecting the tinfected-Sf9 cells and supernatant.

### 2.3. Expression and Purification of SUDV GP and VP40 Antigens and Preparation of Polyclonal Antibodies

The SUDV GP (158~368 aa) and VP40 proteins were generated by a prokaryotic expression system. Briefly, the synthesized genes encoding the SUDV GP (158–368 aa) and VP40 proteins were separately inserted into the prokaryotic expression vector PET 30a (+) by using BamHI and NotI, in-frame with the 6×His-tag on the C- and N-terminus to construct PET 30a (+) -GP-his-C/N and PET 30a (+) -VP40-his-C/N. After the recombinant plasmids were confirmed by restriction digest, the two recombinant plasmids were transformed into *E. coli* BL21(DE3) (Transgen Biotech, Beijing, China) and the transformants selected on Luria-Bertani (LB) agar plates with 100 μg/mL kanamycin. A single clone was picked and inoculated into 4 mL of LB medium with 100 μg/mL kanamycin at 37 °C for overnight growth. Expression was induced with the addition of 0.4 mM IPTG when the optical density at 600 nm (OD_600_) reached 0.6. The proteins were purified using a HisPurTM Ni-NTA Spin Purification kit (Thermo Scientific, Carlsbad, CA, USA) according to the manufacturer’s instructions. To visualize the expression of the purified proteins, samples were resolved on a 12% SDS-polyacrylamide gel (SDS-PAGE) and stained with Coomassie blue, and protein concentrations were determined using a bicinchoninic acid (BCA) assay (Thermo Scientific, Carlsbad, CA, USA) followed by analysis at an absorbance of 570 nm; bovine serum albumin (BSA; Sigma-Aldrich, St. Louis, MO, USA) was used as the protein standard.

Polyclonal antisera against SUDV GP (158~368 aa) or VP40 were prepared by immunizing BALB/c mice with 10 μg purified GP or VP40 recombinant proteins twice at 2-week intervals, and harvesting the mouse serum, which were mouse polyclonal antisera against SUDV GP (158~368 aa) or VP40.

### 2.4. Immunofluorescence Testing of the Recombinant Baculoviruses

The Sf9 cells were infected with the fourth generation recombinant baculovirus rBac-GP-GP or rBac-VP40-VP40 for approximately 48 h, infected Sf9 cells were fixed with 4% paraformaldehyde for 15 min at the room temperature. Infected-Sf9 cells were washed with PBS and blocked with PBS containing 5% BSA (Sigma-Aldrich, St. Louis, MO, USA). Then, the cells were incubated with a 1:100 dilution of a mouse polyclonal antisera against SUDV GP or a mouse polyclonal antisera against SUDV VP40 (generated in our lab) for 1 h at 37 °C, and then washed with PBS and incubated with FITC-conjugated goat anti-mouse IgG (Bioss antibodies, Beijing, China) containing 1% diluted Evans blue for 1 h at 37 °C; then the infected Sf9 cells were washed with PBST (containing 0.05% Tween-20) three times and were observed with a fluorescence microscope. The control cells were incubated with the two primary antisera at the same time. Baculovirus titers were determined using a BacPAKTM Baculovirus Rapid Titer kit (TaKaRa, Dalian, China).

### 2.5. VLP Preparation 

To generate SUDV VLPs, Sf9 cells were co-infected with recombinant baculoviruses rBV-GP-GP and rBV-VP40-VP40 at different ratios of 1:1, 1:2, 1:2.5, 1:3, 2:1, 2.5:1, or 3:1. The SUDV VLPs were harvested at 4-d post-infection. To purify the VLPs, culture supernatants were harvested and spun at 2000× *g*. The crude VLPs were then concentrated by ultracentrifugation at 30,000× *g* for 1 h, and the pellets were resuspended in PBS before purification with a 10–30–50% discontinuous sucrose gradient. Bands between 30–50% sucrose were collected and resuspended in endotoxin-free PBS, and the VLPs concentrations were determined using a BCA assay (Thermo Scientific, Carlsbad, CA, USA) followed by analysis at an absorbance of 570 nm. The VLP preparations were not tested for endotoxin after production.

### 2.6. Western Blotting and Transmission Electron Microscopy (TEM) Analysis of VLPs

Purified SUDV VLPs were processed and examined by Western blotting and transmission electron microscopy. For Western blotting, aliquots containing 10 μg of total protein were diluted with reducing buffer and denatured by heating at 95 °C for 10 min. Proteins were separated in 12% acrylamide gels, before they were transferred onto polyvinylidene fluoride (PVDF) membranes (Merck Millipore, Darmstadt, Germany) under denaturing conditions. For protein detection, two polyclonal antisera were used: mouse anti-SUDV GP polyclonal antisera and mouse anti-SUDV VP40 polyclonal antisera were mixed at a dilution of 1:1500 as a primary antibody for blotting; a goat anti-mouse IgG HRP-conjugated antibody (Bioss antibodies, Beijing, China) was used at a dilution of 1:5000 as a secondary antibody. 

Negative staining transmission electron microscopy (TEM) was used to analyze the shape and size of purified SUDV VLPs. In short, 30 μL of sucrose gradient-purified SUDV VLPs were fixed for 15 min on carbon-coated formvar grids, grids were washed with 30 μL PBS and then treated with 1% phosphotungstate acid for 5 min. Grids were left to air dry and observed by using a HITACHI H-7650 transmission electron microscope.

### 2.7. Animal Immunizations

A total of two batches of BALB/c mice (6 weeks old, female) were purchased from the Changchun Yisi Laboratory Animal Technology Co., Ltd. (Changchun, China) and immunized. In batch one, 24 mice were randomized into four groups (*n* = 6 per group) and were vaccinated intramuscularly. Mice in group one were vaccinated with PBS, mice in group two were vaccinated with Montanide ISA 201 VG (ISA 201) adjuvant (Seppic, Paris, France), mice in group three were vaccinated with 20 μg of SUDV VLPs-only, and mice in group four were vaccinated with 20 μg of SUDV VLPs mixed with an equal volume of Montanide ISA 201 VG (ISA 201) adjuvant, and all groups were vaccinated twice at 3-week intervals (Figure 5A). The mouse sera were collected at two-weeks after every immunization. One week after the booster immunization, splenocytes from 3 mice of each group were isolated. In batch two, nine mice were randomly distributed into three groups (*n* = 3 per group) (PBS group, ISA 201 adjuvant, 20 μg of SUDV VLPs mixed with an equal volume of ISA 201 adjuvant) and were vaccinated intramuscularly. One week after the primary immunization, the inguinal lymph nodes were collected from 3 mice.

Two healthy male horses (numbered #392 and #18), 2–6 years old, 400–500 kg in weight and without detectable antibodies against SUDV detected by indirect ELISA, were supplied by Red Hill Military horse farm. The horses were multipoint injected subcutaneously in the rear area with 1.0, 2.0, 3.0, 3.0, or 4.0 mg of purified SUDV-VLPs for a total of 5 times, primary immunization mixed with an equal volume of Freund’s incomplete adjuvant (Thermo Scientific, Rockford, IL, USA) and booster immunization mixed with an equal volume of Freund’s complete adjuvant, maximum immune volume does not exceed 4 mL, with boosting at two week intervals (Figure 5B). The horse sera were collected before each immunization and stored at −20 °C for further studies.

### 2.8. Detection of SUDV GP-Specific Antibody by ELISA 

The mice and horse serum samples were collected two weeks after each immunization. Levels of SUDV GP-specific antibodies were detected by indirect ELISA. Briefly, 100 μL of purified -prokaryotic expressed SUDV GP was coated on ELISA plates at a concentration of 10 µg/mL overnight at 4 °C, and then the plate was blocked with 2% BSA for 2 h at 37 °C. The serum was added (100 μL/well), and it was 2-fold serially diluted in 2% BSA; plates were subsequently incubated for 1.5 h at 37 °C, and 100 μL of HRP-labeled goat anti-mouse IgG or HRP-labeled goat anti-horse IgG (Bioss antibodies, Beijing, China), diluted 1:10,000 in 2% BSA was added to each well. After one hour of incubation at 37 °C, 100 μL of 3,3′,3,5′-tetramethylbenzidine (TMB) (Sigma-Aldrich, St. Louis, MO, USA) substrate solution was added to each well and then was stopped with the addition of 50 μL of 0.5 M H_2_SO_4_. Optical density (OD) values were measured at a wavelength of 450 nm (OD_450_). After each incubation step, ELISA plates were washed five times with PBST. Mean OD values were considered positive if the OD values were more than two times the value of the negative control.

### 2.9. Detection of SUDV Neutralizing Antibodies by a Pseudotyped Virus

A pseudotyped virus neutralization assay was performed to test the neutralzing antibodies. Recombinant lentiviral vectors that express SUDV glycoprotein and carry a luciferase reporter gene were produced as described previously [31], with some modifications. Briefly, 239T cells were seeded in 60-mm culture dishes (Corning, NY, USA), and 24 h later, the 80% subconfluent cells were co-transfected with 3 μg of pcDNA 4.0-GP (the glycoprotein expression vector) and 3 μg of pNL4-3.Luc. RE vector by Lipofectamine^TM^ 3000 Transfection Reagent (Thermo Scientific, Carlsbad, CA, USA). After 2 days, the supernatant containing the pseudotyped viruses was harvested, and the titer was determined in Huh7 cells as previously described [32]. For testing of neutralizing antibodies, 2-fold serially diluted serum samples were mixed with 100 TCID_50_ of pseudotyped viruses for 0.5 h at 37 °C and were then added to Huh7 cells. After 4 h, the inoculum was removed and replaced with fresh media. Then, cells were lysed at 48 h with cell lysis buffer, that was followed by the addition of 100 μL of luciferase substrate (Promega, Madison, WI, USA) to determine luciferase activity. The luciferase activity of the samples was measured with the Infinite M200 Microplate Spectrophotometer (Tecan, Mannedorf, Switzerland). The percent inhibition rate was calculated as previous showed [1]. The experiments were independently repeated three times.

### 2.10. Cell-Mediated Immune Responses in Mice

One week after the booster immunization in batch one, three mice from each group were randomly selected and euthanized. Their spleens were harvested into a tissue culture dish, and each spleen was roughly minced and pressed through a 5-mL syringe. The cells were filtered through a 40-μm filter (BD Falcon 40-μm strainer) and centrifuged at 2000 rpm for 10 min at the room temperature. The cells were processed by resuspension in a red blood cell lysis buffer and centrifuged at 2000 rpm for 10 min at twice the room temperature. The splenocytes were harvested and washed with RPMI 1640 medium (Gibco, San Diego, CA, USA) containing 10% FBS (Gibco, San Diego, CA, USA). Then, the splenocytes were cultured in RPMI 1640 medium containing 10% FBS and stimulated with or without the purified SUDV-GP antigen (10 μg/mL). Following incubation at 37 °C in 5% CO_2_ for 48 h, the frequencies of splenocytes producing IFN-γ or IL-4 were measured using mouse ELISpot kits (Mabtech AB, Stockholm, Sweden) according to the manufacturer’s instructions. Spot-forming cells (SFCs) were enumerated by an automated ELISpot reader (AID ELISPOT reader-iSpot, Germany).

The levels of cytokines in the supernatant of stimulated splenocytes were detected by commercial ELISA. Splenocytes were stimulated as described above and were then incubated for 48 h. The cell culture supernatant was collected by centrifugation at 3000 rpm for 15 min, and the manufacturer directions were followed to detect IL-2, IL-4, IL-10, IFN-γ, or TNF-α by ELISA kits (Mabtech AB, Stockholm, Sweden).

### 2.11. SUDV VLPs Induce Activation of B cells

Inguinal lymph node samples were harvested from batch two vaccinated mice 7 days after the primary immunization. Lymphoid cells were harvested into a tissue culture dish, and were roughly minced and pressed through a 5 mL syringe. The cells were filtered through a 40-μm filter (BD Falcon 40-μm strainer). Then, prepared in PBS with 2% FBS and were stained with equal volumes of 1:250 dilutions of anti-CD19-APC and anti-CD40-FITC antibodies (BD Biosciences, Franklin, VA, USA) for 30 min at 4 °C; the labeled B cells were then washed twice with PBS with 2% FBS and analyzed using a FACSAria TM Cell Sorter (BD Biosciences, Franklin, VA, USA).

### 2.12. Horse Immunoglobulin Purification

For equine antisera, the blood was taken from the jugular vein of #392 immunized horses, and the sera were collected. The horse serum was diluted 8-fold with PBS and then added to a saturated ammonium sulfate solution until the ammonium sulfate concentration was 50%. The solution was allowed to stand at 4 °C for 3 h, and it was centrifuged at 5000 rpm for 20 min. After removing the supernatant, the precipitate was dissolved in PBS, and saturated ammonium sulfate was added until the concentration of ammonium sulfate was 33%. The solution was allowed to stand at 4 °C for 3 h, and centrifuged at 5000 rpm for 20 min. The precipitate was dissolved in PBS and dialyzed against PBS at 4 °C for 18 h to remove the ammonium salt.

### 2.13. Laboratory Facility and Ethics Statement

The treatment of all mice was in accordance with the Welfare and Ethical guidance of Laboratory Animals of China (GB 14925-2001). The agreement was approved by the Animal Welfare and Ethics Committee of the Institute of Veterinary Medicine of the Military Academy of Sciences (Laboratory Animal Care and Use Committee Authorization, permit number JSY-DW-2018-02).

## 3. Results

### 3.1. Expression of SUDV GP and VP40 Proteins

**Figure 1:**
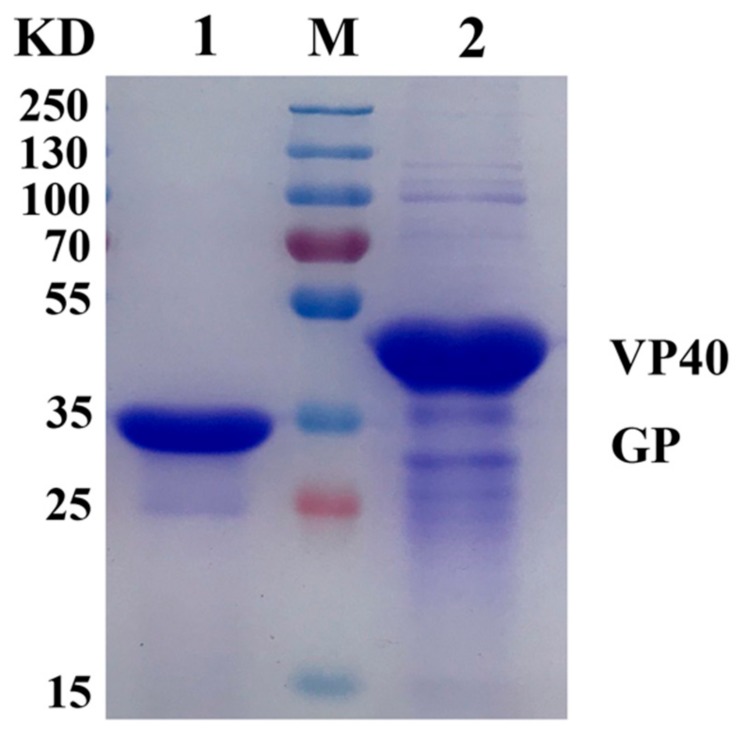
SDS-PAGE analysis of purified SUDV GP and VP40 proteins. Prokaryotic-expressed SUDV GP (158–368 aa) and VP40 proteins were purified by 6×His-tag affinity chromatography and detected by Coomassie-stained gel. The Lane 1 is the purified SUDV GP protein (32 kDa) and lane 2 is the purified SUDV VP40 protein (40 kDa), M is the protein molecular marker.

The sequences encoding the GP (158–368 aa) and VP40 proteins of SUDV were cloned into the prokaryotic expression vector PET 30a (+), resulting in the plasmid PET 30a (+) -GP-his-C/N and PET 30a (+) -VP40-his-C/N (C and N terminal 6×His-tag). Correct insertion of the sequences in the recombinant plasmid was confirmed by restriction digest mapping analysis and DNA sequencing. The recombinant plasmids PET 30a (+) -GP-his-C/N and PET 30a (+) -VP40-his-C/N were transformed into *E. coli* BL21(DE3) cells. Recombinant proteins SUDV GP and VP40 were experessed in cells after added 0.4 mM IPTG. The proteins were purified by 6×His-tag affinity chromatography and detected by SDS-PAGE analysis (Figure 1). The purpose of expressing SUDV GP and VP40 proteins is to prepare anti-GP polyclonal antisera and anti-VP40 polyclonal antisera, and these two polyclonal antisera were successfully made through immunized mice with these two proteins twice respectively.

### 3.2. Verification of Baculovirus-Expressed SUDV GP and VP40 Proteins

**Figure 2:**
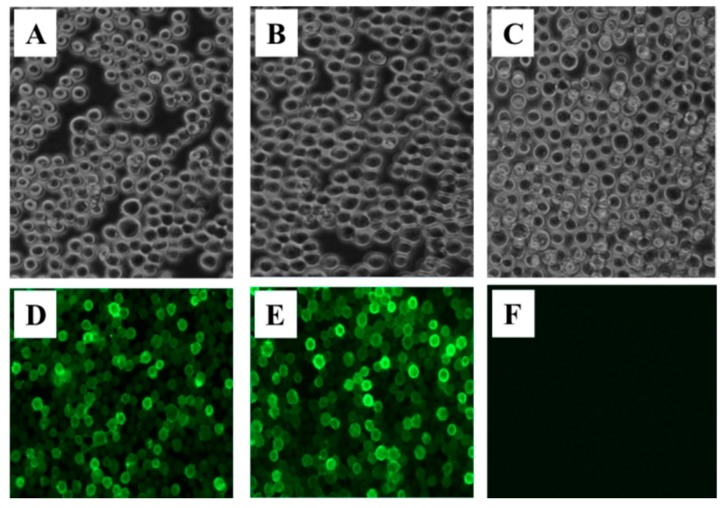
Immunofluorescence assay confirms the expression of SUDV GP and SUDV VP40 proteins in Sf9 cells (magnification of microscopy images, 200×). Recombinant baculovirus infected Sf9 cells became larger and rounder (**A**,**B**). Anti-SUDV GP and anti-SUDV VP40 polyclonal antisera were used to detect respectively the expression of SUDV GP and VP40. A specific green fluorescent signal around the GP-expressing (**D**) and VP40-expressing (**E**) cells indicates that these two proteins are expressed. The mock cells (**C**) were incubated the two polyclonal antisera showed negative staining (**F**).

To identify the successful rescue of the recombinant baculovirus and effectively expressed the SUDV GP and VP40 proteins, the fourth generation of recombinant baculovirus were detected by IFA. Sf9 cells infected with recombinant baculovirus rBac-GP-GP or rBac-VP40-VP40 were incubated with mouse anti-GP polyclonal or anti-VP40 polyclonal antisera respectively, and then incubated with FITC-conjugated goat anti-mouse IgG. The immunofluorescence results showed that Sf9 cells infected with recombinant baculovirus rBac-GP-GP or rBac-VP40-VP40 were shown in specific green (Figure 2D,E) and non-infected Sf9 cells (Figure 2F) did not show, which means that SUDV GP and VP40 proteins were successfully expressed in infected Sf9 cells.

### 3.3. Production and Characterization of SUDV VLPs

**Figure 3:**
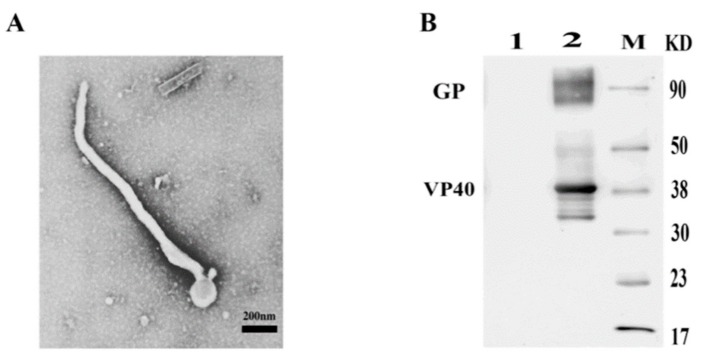
Characterization of the SUDV VLPs. Representative electron microscopy image of the SUDV VLPs; scale bar = 200 nm (**A**). Western blot analyses of SUDV GP and SUDV VP40 in purified SUDV VLPs by incubating with mouse anti-SUDV GP polyclonal antisera and mouse anti-SUDV VP40 polyclonal antisera at the same time. Lane 1 is the uninfected-Sf9 cells and lane 2 is the purified SUDV VLPs, M is the protein molecular marker (**B**).

SUDV VLPs were generated by co-infecting with recombinant baculoviruses rBV-GP-GP and rBV-VP40-VP40 in Sf9 cells. The SUDV VLPs were harvested from the culture supernatant and purified as described in the Material and Methods. To further characterize the structure of the SUDV VLPs, we examined purified material by transmission electron microscopy. A negative staining study revealed that the SUDV VLPs were found to mimic the naive virus in structure and size; they were approximately 80 nm in diameter and 800–1500 nm in length (Figure 3A).

The identity of the protein component of the purified SUDV VLPs was assessed by Western blot analysis with mouse anti-SUDV GP polyclonal antisera and mouse anti-SUDV VP40 polyclonal antisera. Both VP40 and GP proteins are present in the VLPs (Figure 3B). These results demonstrate that SUDV GP and VP40 efficiently assembled SUDV VLPs and released by insect cells.

### 3.4. SUDV VLPs Elicit Antibody Responses in Vaccinated Mice

**Figure 4:**
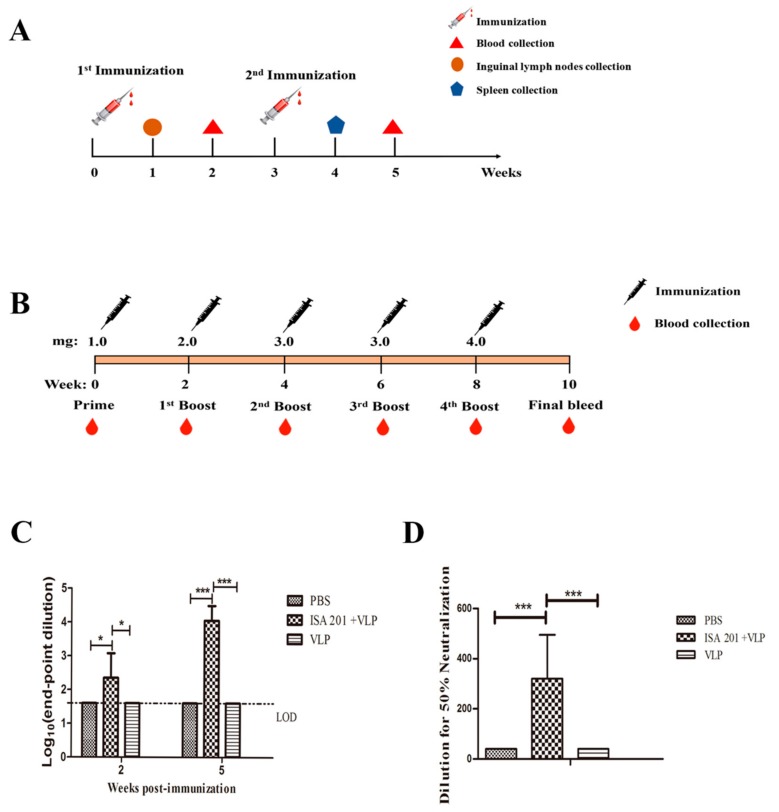
Mice and horse immunization procedure, analysis of SUDV GP-specific antibody, and neutralizing antibodies in vaccinated mice. The immunizations of mice (**A**) or horses (**B**). Analysis of SUDV-VLP-induced specific IgG antibody response by indirect ELISA at two weeks after every immunization of vaccinated mice (**C**). SUDV neutralizing antibody titers are detected in immunized mice at two weeks after booster immunization (**D**). Limit of detection (LOD) means the minimum concentration or content that can be detected under the determined experimental conditions. Error bars represent the standard deviation. The *p-*values were determined according to a Tukey’s multiple comparison test (* *p* < 0.05, *** *p* < 0.001).

To detect a SUDV-VLP-induced humoral immune response in vaccinated BALB/c mice, indirect ELISA and virus neutralization assay were performed to evaluate the production of SUDV GP-specific antibodies and neutralizing antibodies. At the fifth week of the immunization, the sera from mice treated with 20 μg SUDV VLPs mixed with an ISA 201 adjuvant group had high titers of SUDV specific IgG antibodies with endpoint titers up to 1:81,920 (Figure 4C), virus-neutralizing antibody titers averaged 1:320 and individual mice neutralizing antibody titers were up to 1:640 (Figure 4D), showing significant divergence when compared to the PBS group and ISA 201 adjuvant group. But the SUDV specific IgG antibodies and neutralizing antibodies were not detected in the VLPs-only group mice. These results demonstrate that SUDV VLPs at 20 μg antigen dose cannot stimulate the production of antibodies in mice, but this antigen dose mixed with ISA201 adjuvant can stimulate mice to produce specific IgG antibodies and neutralizing antibodies.

### 3.5. Antigen-Specific Cellular Immune Responses in Vaccinated Mice 

**Figure 5:**
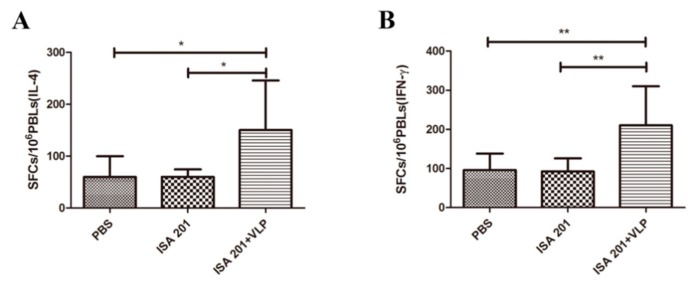
IFN-γ and IL-4 secretion by proliferating splenic induced by a purified baculovirus-expressed SUDV GP protein. Splenocytes from immunized mice and were stimulated with purified -prokaryotic expressed SUDV GP (10 μg/mL) for 48 h, and the level of IL-4 (**A**) or IFN-γ (**B**) were quantitated using an ELISpot assay. The data represent the mean ± standard deviation (SD) of SFCs per million cells. Statistical analysis between the two groups was analyzed by using Tukey’s multiple comparison test (* *p* < 0.05, ** *p* < 0.01).

ELISpot assays were used to evaluate the antigen-specific cellular immune response in vaccinated mice. Since 20 μg VLPs-only did not stimulate the production of specific antibodies and neutralizing antibodies, the VLPs-only group was removed in the cellular immune response experiment. The results from the IL-4 ELISpot assay are shown in Figure 5A. The SFCs produced from the splenocytes of mice immunized with 20 μg SUDV VLPs mixed with an ISA 201 adjuvant were significantly higher compared with mice immunized with PBS or ISA 201. The immunization with 20 μg SUDV VLPs mixed with an ISA 201 adjuvant also resulted in increased IFN-γ responses, with SFCs production that were significantly higher than that following immunization with PBS or ISA 201 (Figure 5B). These results demonstrate that 20 μg SUDV VLPs mixed with an ISA 201 adjuvant enhances IL-4 and IFN-γ responses in mice.

### 3.6. SUDV VLPs-Enhanced Splenocytes Cytokine Secretion

**Figure 6:**
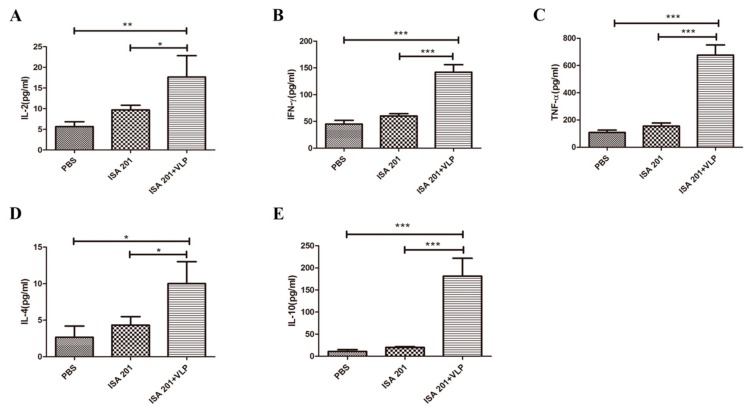
Detection of cytokine secretion in splenocytes. Splenocytes were collected from mice one week after following the booster immunization and were stimulated with purified-prokaryotic expressed SUDV GP protein for 48 h. The level of IL-2 (**A**), IFN-γ (**B**), TNF-α (**C**), IL-4 (**D**), and IL-10 (**E**) in the supernatant were measured with commercial ELISA kits (*n* = 3). Data are shown as the means ± SDs and were analyzed by using Tukey’s multiple comparison test (* *p* < 0.05, ** *p* < 0.01, *** *p* < 0.001).

To further investigate the antigen-specific cellular immune responses induced by SUDV VLPs, cytokines secreted by splenocytes were measured by commercial ELISA kits. Splenocytes were harvested from mice one week after the booster immunization and stimulated with Prokaryotic-expressed SUDV GP (158~368 aa). Cytokines secreted into the supernatant by splenocytes were assessed. Type 1 cytokines such as IL-2 (Figure 6A), IFN-γ (Figure 6B), and TNF-𝛼 (Figure 6C) were significantly higher in the group immunized with 20 μg SUDV VLPs mixed with an ISA 201 adjuvant than they were in the PBS and ISA 201 adjuvant group. Type 2 cytokines, such as IL-4 (Figure 6D) and IL-10 (Figure 6E), showed the same trend. These results indicate that 20 μg SUDV VLPs mixed with an ISA 201 adjuvant potently enhance cellular immune response in mice.

### 3.7. Enhancing Effects of SUDV VLP on B Cell Activation

**Figure 7:**
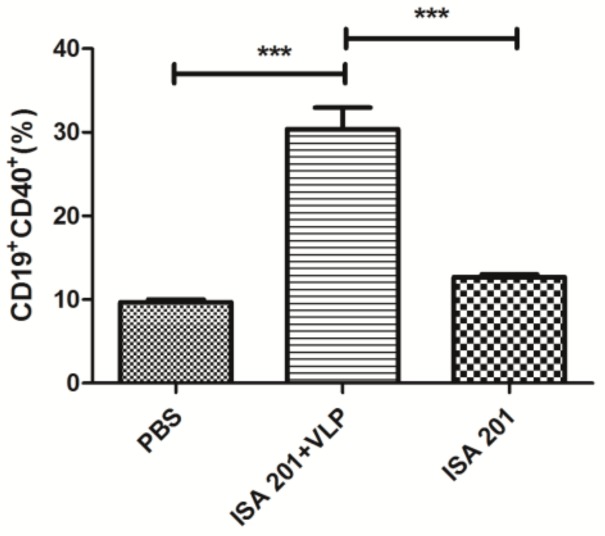
Activation of B cell in BALB/c mice. At seven days after the primary immunization, inguinal lymph nodes were collected from the mice treated with PBS, ISA 201 adjuvant and 20 μg SUDV VLPs mixed with ISA 201 adjuvant, and B cell activation was analyzed by staining with anti-CD19-APC and anti-CD40-FITC antibodies. Data are shown as the means ± SDs and were analyzed by using Tukey’s multiple comparison test (*** *p* < 0.001).

To study whether the vaccine can induce B cell activation, inguinal lymph nodes were collected from the mice vaccinated with PBS, ISA 201 adjuvant and 20 μg SUDV VLPs mixed with ISA 201 adjuvant. The result showed (Figure 7) that the percentage of activated B cells (CD19^+^CD40^+^) was significantly higher in the 20 μg SUDV VLPs mixed with ISA 201 adjuvant group than in the PBS group and ISA 201 adjuvant group.

### 3.8. Antibody Response in Vaccinated Horses

**Figure 8:**
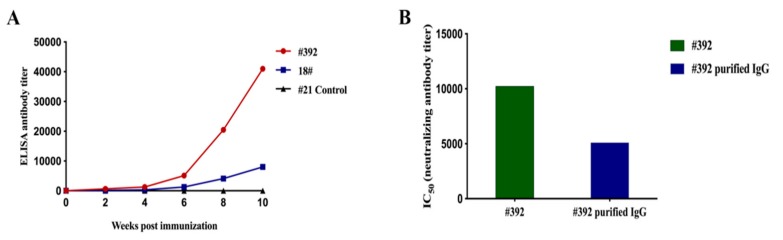
The detection of humoral immune responses in horses after immunized SUDV VLPs mixed with Freund’s adjuvant. (**A**) Measurement of SUDV GP-specific IgG antibody by indirect ELISA and (**B**) detection of neutralizing activity by using pseudotyped viruses.

The humoral immune response in horses was assessed at 2, 4, 6, and 8 weeks after immunization (Figure 4B). Serum samples were collected to measure the SUDV GP-specific antibodies and neutralizing antibody. The level of SUDV GP-specific IgG antibody was detected from the second week (Figure 8A). After the fifth immunization, the titer of SUDV GP-specific IgG antibody of horse #392 was 1:40,960, and for horse #18 was 1:8192. The results revealed that SUDV VLPs mixed with Freund’s adjuvant could stimulate horses to produce humoral immune response. Regarding the research on SUDV therapeutic antibodies, we selected #392 horse serum for further study. The #392 serum was extracted and purified to obtain purified IgG; #392 horse serum and #392 purified IgG were simultaneously subjected to virus neutralization. These findings showed that the neutralizing antibody titer of #392 horse serum was 1:10,240 after the fifth immunization, correspondingly, the neutralizing antibody titer of purified IgG was 1:5120 (Figure 8B).

## 4. Discussion

The mechanism of natural immunity against EBOV remains unclear. The efficacy of different types of vaccines may vary depending on the vaccine platform: antibody is the major immune correlate factor of the rVSV-ZEBOV vaccine [33]. Meanwhile, CD8 T cell responses have been attributed to protection by the Ch-Ad5 vaccine [34]. Previous VLP vaccine studies showed it is efficacious against lethal Ebola challenge, however, with different adjuvants to save antigen and enhance vaccine-induced immune responses [35,36].

The impact of SUDV outbreaks in recent years, and the potential for viral spread to non-endemic regions or countries, makes the development of safe and efficacious vaccines urgent.

Previous findings revealed that SUDV VLPs could be readily assembled by the co-expression of baculoviruses expressing GP, NP and VP40 in insect cells [30]. Here, we demonstrated that co-infection of rBV-GP-GP and rBV-VP40-VP40 recombinant baculoviruses can result in the successful assembly of VLPs in insect cells, the morphology of SUDV VLPs is similar to a native virus, and the immunogenicity of VLPs was tested with an ISA 201 adjuvant as a candidate vaccine in mice.

In our study, we generated recombinant baculaviruses rBV-GP-GP and rBV-VP40-VP40 using the pFastBacDual vector to increase the protein production utilizing the dual promoter. To determine the optimal proportion of two recombinant baculaviruses rBV-GP-GP and rBV-VP40-VP40, we also tried to co-infect Sf9 cells with recombinant baculaviruses rBV-GP-GP and rBV-VP40-VP40 at ratios of 1:1, 1:2, 1:2.5, 1:3, 2:1, 2.5:1, and 3:1. At a ratio of 2.5:1, the morphology of VLPs was similar to the native virus, as assessed by TEM (Figure 3A). The Western blot result (Figure 3B) showed that SUDV GP and VP40 efficiently assembled SUDV VLPs.

The immunization results depicted the failure of the group vaccinated with 20 μg of SUDV VLPs-only to stimulate the production of humoral immune responses in mice, while the group vaccinated with 20 μg of VLPs mixed with an ISA 201 adjuvant could stimulate the production of humoral immunity in mice. Presumably, immunization with 20 μg of SUDV VLPs-only is an insufficiently low dose of an immunogen that cannot stimulate the mice to produce an immune response, and the use of an ISA 201 adjuvant with SUDV VLPs might cause low-dose immunogens to initiate a response, and using with adjuvant can save antigen amount (Figure 4C). The same SUDV VLPs antigen dose (20 μg) mix different adjuvants could stimulate mice to produce different degrees humoral immune responses. The ISA 201 adjuvant showed the most effective adjuvant than other adjuvants in mice. The detection of neutralizing antibody showed that sera from mice immunized with 20 μg SUDV VLPs mixed with ISA 201 adjuvant could neutralize approximately 50% of the pseudotyped viruses at an average 1:320 dilution, while individual samples were effective with as high as a 1:640 dilution (Figure 4D).

Both humoral and cellular responses are indispensable for providing protection. IFN-γ is a Th1-type cytokine involved in the antiviral action of cellular immune responses and IL-4 is mainly produced by Th2 cells and is associated with humoral immune responses. The SUDV VLPs could effectively stimulate Th1 and Th2 cytokine production in vaccinated mice (Figure 5A,B). The levels of cytokines secreted from splenocytes, such as IL-2, IFN-γ, and TNF-α secreted from Th1 cells (Figure 6A–C) and IL-4 and IL-10 were secreted from Th2 cells (Figure 6D,E), were significantly increased after vaccination. Moreover, our study revealed that the B cells of mice of the group vaccinated with SUDV VLPs mixed with an ISA 201 adjuvant were activated at one week after the primary immunization (Figure 7).

In addition, we have conducted research on therapeutic antibodies with the goal of preparing antibodies for the post-exposure treatment of SUDV. Horses were selected as immunized animals in our study because horse anti-immunoglobulins have been used in the treatment of various viral infections [37,38,39]. SUDV VLPs can effectively induce humoral immune responses in horses after immunization (Figure 8A). The horse anti-SUDV immunoglobulin was obtained from #392 horse serum through crude extraction and purification. The neutralizing antibody titer of purified #392 IgG was 1:5120, which was one time lower than that before purification (Figure 8B).

We tested the SUDV VLPs immunogenicity and neutralizing activity of hours purified IgG by using pseudo typed viruses, and efficacy studies still need to be performed. These SUDV VLPs vaccine and horse purified IgG provide ideas for the development of vaccines and therapeutic antibodies that could prevent and treat SUDV infections. The Ebola epidemic is still spreading, vaccination is an effective means of preventing and controlling the outbreaks, and effective antibodies represent key drugs for the treatment of Ebola patients. Therefore, further Ebola vaccine and therapeutic antibody research are needed.

## Supplementary Materials

The following are available online at https://www.mdpi.com/1999-4915/12/1/64/s1, Figure S1: Diagram of the plasmids encoding the pFastBacDual-GP-GP (A) and pFastBacDual-VP40-VP40 (B).
