# Supplementary material for: A Chimeric Sudan Virus-Like Particle Vaccine Candidate Produced by a Recombinant Baculovirus System Induces Specific Immune Responses in Mice and Horses
Source: Viruses. 2020 Jan 3;12(1):64. doi: 10.3390/v12010064 (PMC7019897; doi:10.3390/v12010064)

Supplementary Materials:

The following are SUDV GP and VP40 sequences used in SUDV VLPs:

VP40 sequence:

ATGAGGAGGGTTACCGTTCCTACTGCTCCACCTGCTTACGCTGATATCGGATACCCTATGAGCATGCTG
CCAATCAAGTCCAGCCGCGCTGTTTCCGGTATCCAACAGAAGCAGGAAGTCCTCCCTGGAATGGATAC
CCCATCCAACTCCATGAGACCTGTGGCTGATGACAACATCGACCACACCTCCCACACACCTAACGGA
GTTGCTTCCGCTTTCATCCTGGAAGCTACCGTTAACGTGATCAGCGGTCCAAAGGTCCTCATGAAGCA
GATCCCTATCTGGCTGCCACTCGGAATCGCCGATCAGAAGACCTACAGCTTCGACAGCACCACAGCTG
CCATTATGTTGGCCAGCTACACCATCACCCACTTCGGAAAGGCCAACAACCCATTGGTTCGTGTTAAC
CGTCTGGGTCAGGGTATTCCAGACCACCCATTGCGTTTGCTCCGCATGGGAAACCAGGCCTTTTTGCAG
GAATTCGTGCTGCCACCAGTGCAATTGCCACAGTACTTCACTTTCGACCTGACTGCTCTCAAGCTCGTG
ACACAGCCTCTCCCTGCCGCCACATGGACAGATGAAACTCCTTCAAACCTGAGTGGTGCCCTGCGTCC
TGGTCTGTCATTTCACCCTAAGCTGCGCCCTGTCCTGCTGCCCGGCAAAACTGGCAAAAAAGGTCATG
TCTCTGATCTGACTGCCCCCGACAAGATTCAAACAATCGTGAATCTGATGCAAGACTTCAAGATCGTG
CCCATCGACCCCGCCAAGTCTATCATCGGCATCGAGGTGCCCGAGTTGCTGGTCCATAAGCTCACAGG
CAAAAAGATGTCACAAAAGAATGGCCAACCCATCATCCCCGTCCTCCTGCCCAAGTACATCGGCCTC
GACCCCATTTCTCCCGGCGACCTCACTATGGTCATTACACCCGACTATGACGACTGTCACAGTCCCGCC
TCTTGCTCTTACCTCTCAGAGAAGTAA

GP sequence:

ATGGGTGGTTTGAGCTTGCTCCAACTCCCAAGAGACAAGTTCCGCAAGAGCTCCTTCTTCGTGTGGGTC
ATCATCCTGTTCCAAAAGGCTTTCTCCATGCCTCTGGGTGTCGTGACCAACAGCACCCTGGAGGTTACC
GAGATCGACCAACTGGTCTGTAAGGACCACTTGGCTAGTACCGATCAGTTGAAGTCCGTGGGTTTGAA
CCTCGAGGGTAGTGGTGTGTCTACCGACATCCCTTCCGCTACCAAGAGATGGGGATTCAGGTCCGGTG
TTCCTCCTAAGGTTGTTAGCTACGAGGCTGGAGAATGGGCCGAAAACTGCTACAACTTGGAGATCAAG
AAGCCTGACGGTAGCGAGTGCTTGCCACCCCCACCAGACGGTGTTCGTGGATTTCCAAGATGCAGGTA
CGTTCACAAGGCTCAAGGAACCGGTCCATGTCCAGGTGACTACGCTTTCCATAAGGACGGAGCTTTCT
TCCTCTACGACAGGTTGGCTTCCACCGTTATCTACAGGGGTGTGAACTTCGCTGAGGGAGTGATCGCTT
TCCTCATCCTCGCTAAGCCAAAGGAGACATTCCTCCAGTCCCCACCAATCAGGGAAGCTGTGAACTAC
ACCGAAAACACCTCCAGCTACTACGCTACCTCCTACCTGGAATATGAGATCGAGAACTTCGGCGCCCA
GCACTCCACCACATTGTTCAAGATCGACAACAACACCTTCGTCCGTCTGGACCGCCCACATACCCCTC
AATTCCTCTTCCAGCTGAACGACACCATCCACTTGCACCAGCAACTCTCCAACACCACCGGACGTTTG
ATCTGGACCCTGGACGCTAACATCAACGCTGACATCGGTGAGTGGGCTTTCTGGGAAAACAAGAAGA
ACCTCTCCGAGCAGCTGCGTGGAGAGGAACTCAGCTTTGAGGCTCTGTCTTTGAACGAGACCGAAGAC
GATGATGCTGCTTCTAGCCGTATCACCAAGGGACGTATCAGCGATAGGGCTACAAGGAAGTACAGCG
ACCTGGTTCCTAAGAACAGCCCAGGTATGGTGCCACTGCATATCCCAGAGGGAGAAACAACCCTCCC
TTCTCAAAACAGCACAGAGGGTCGTCGTGTTGGTGTGAATACCCAAGAAACCATCACCGAGACAGCC
GCCACAATCATCGGAACTAACGGAAACCACATGCAGATCTCAACAATTGGCATCCGTCCTAGTTCATC
TCAGATCCCTAGTTCTTCACCTACAACTGCCCCCTCACCTGAGGCTCAGACACCCACAACACACACAT
CAGGTCCCTCTGTCATGGCCACAGAGGAACCCACTACTCCCCCTGGATCATCTCCCGGCCCAACTACT
GAAGCCCCTACTCTCACAACACCCGAAAATATCACTACAGCCGTGAAGACTGTCCTGCCCCAAGAAT
CTACTTCAAACGGCCTGATCACTTCAACAGTCACTGGCATCCTCGGCAGTCTCGGCCTCCGTAAGCGTT
CACGCCGCCAAACTAACACAAAGGCCACTGGCAAATGTAACCCCAACCTGCACTACTGGACTGCCCA
AGAACAACACAACGCCGCCGGCATTGCCTGGATTCCCTATTTGGCCCCGGTGCCGAAGGCATTTACA

CTGAAGGCCTGATGCACAATCAGAATGCCCTCGTCTGTGGACTCCGCCAGCTCGCCAATGAAACTACT
CAGGCCCTGCAGCTCTTTCTGCGCGCCACAACTGAACTGCGCACTTACACAATTCTCAATCGCAAAGC
CATTGATTTTCTGCTGCGCCGCTGGGGCGGCACATGTCGCATTCTGGGCCCCGATTGCTGCATTGAACC
CCACGATTGGACAAAAAACATCACTGATAAAATCAACCAGATCATCCACGACTTCATCGACAATCCC
CTGCCTAACCAGGACAACGATGATAATTGGTGGACTGGCTGGCGTCAGTGGATCCCTGCCGGCATCGG
CATCACTGGAATCATTATCGCCATCATTGCCCTGCTGTGTGTCTGCAAACTGCTGTGCTAA

Figure S1. Diagram of the plasmids encoding the pFastBacDual-GP-GP (A) and pFastBacDual-VP40-VP40.

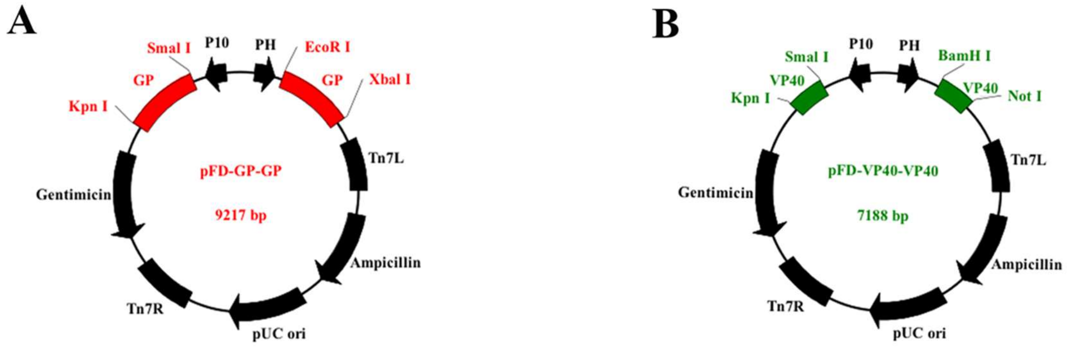

Supplement: Supplementary file 1 [file viruses-12-00064-s001.pdf]
